# Detergent Virus Inactivation in Chromatography Systems

**DOI:** 10.1002/bit.70231

**Published:** 2026-05-06

**Authors:** Kang Cai, Etienne Utiger, Chris Afdahl, Rayan Zamat, Jennifer Anderson, Yeldys De Armas, Gisela Ferreira

**Affiliations:** ^1^ Biopharmaceutical Development AstraZeneca Maryland USA

**Keywords:** chromatography, detergent, protein purification, virus inactivation

## Abstract

Detergent treatment is a widely utilized virus‐inactivation step in therapeutic protein manufacturing to safeguard products. Traditionally, this operation is performed in an incubation vessel in batch mode. In this investigation, a methodology was developed to enable virus inactivation via a post‐load, detergent‐containing wash within a bind‐elute chromatography process. Application of the non‐ionic detergent Laureth 9 during the post‐load wash achieved more than 4 logs of retrovirus inactivation. Chromatography control experiments conducted without detergent resulted in negligible virus inactivation. Simultaneous measurements of virus infectivity and genome copies distinguished the contributions of detergent‐driven net virus inactivation from those of separation‐driven net virus removal. These results establish a robust and simplified alternative approach for virus inactivation in therapeutic protein manufacturing.

## 1 Introduction

In recombinant therapeutic protein manufacturing, a virus inactivation step is typically included during purification to safeguard against potential contamination by enveloped viruses originating from cell culture. Low pH inactivation is a common method that denatures virus envelope proteins, but it requires cumbersome titrations, an incubation step in a large vessel, and may not be tolerated by some protein products (Brorson et al. 2003). Non‐ionic detergents inactivate virus by deforming the envelope membrane and solubilizing envelope proteins, with little impact on the protein product (Horowitz et al. 1998; Dichtelmüller et al. 2009; Hunter et al. 2022; Du and Wu 2026; Luo et al. 2020). This was recently confirmed using time‐lapse total internal reflection fluorescence, which demonstrated deformation of individual virus upon detergent contact (Negi et al. 2024). The non‐ionic detergent Laureth 9 (L9) was used in this investigation. It is a compendial material (USP‐NF) with a proven safety profile, as the same chemical entity serves as the active ingredient in an injectable human medicine (Asclera) (Chemische Fabrik Kreussler & Co. GmbH 2022).

**Figure 1:**
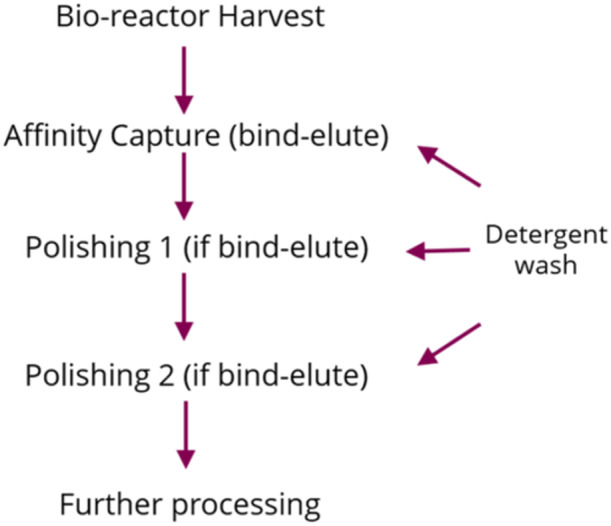
Detergent virus inactivation can be applied as a wash step to any bind‐elute chromatography step during protein purification, including affinity capture and bind‐elute polishing steps.

In a typical therapeutic protein purification process (Figure 1), the affinity‐based capture and one of the polishing chromatography steps are operated in bind‐elute mode. This setup offers opportunities to incorporate a post‐load detergent wash to effectively inactivate potential contaminating enveloped viruses.

The detergent wash option has been previously explored due to its operational simplicity—it only requires adding detergent to an existing wash buffer to achieve virus inactivation. Using the detergent lauryldimethylamine oxide (LDAO) in wash buffer for Protein A affinity chromatography, Bolton et al. demonstrated greater than 6 logs of combined removal and inactivation of xenotropic murine leukemia virus (XMuLV), a commonly used model retrovirus to represent endogenized provirus in cell substrate genome (Shepherd et al. 2003). However, only 1 log can be attributed to net inactivation, as removal contributed about 5 logs (Bolton et al. 2015). Polasek et al. demonstrated on‐column virus inactivation using the traditional solvent/detergent combination of polysorbate 80/tri‐*n*‐butyl phosphate (PS80/TNBP) wash during bind‐elute weak anion exchange chromatography (Polasek et al. 2023). However, the reported virus reduction suggests confounded virus inactivation and removal mechanisms, rather than distinguishing between the net inactivation versus net removal capacities. Using a pH 4.4 acidic wash buffer, Roberts et al. demonstrated 6.5 logs of combined virus removal and inactivation for a copper chelate affinity chromatography process, of which approximately 2 logs were attributed to net inactivation based on free virus inactivation in the pH 4.4 solution (Roberts et al. 1994). While the measured extent of virus inactivation was somewhat limited, these earlier investigations suggested that resin‐bound viruses can be potentially inactivated on column, by detergent or low pH.

In a chromatography setting, the spiked virus in the load can be distributed among the flowthrough, wash, product, and strip fractions. Therefore, virus removal and inactivation mechanisms are intertwined when comparing the virus in the load to the product. To elucidate the net contribution of virus inactivation alone, virus infectivity and genome copy number (gc) were measured for samples from all the abovementioned chromatography fractions. The infectivity readout was normalized against the virus gc in the sample to exclude the contribution of virus removal. Using this methodology, we demonstrate virus inactivation occurring over the population of resin‐bound viruses (stationary phase), an aspect not explicitly addressed in current literature. Additionally, control chromatography experiments conducted without detergent confirmed that any observed virus inactivation was definitively caused by the detergent.

Virus inactivation using L9 wash was effective in both affinity chromatography (AC) and cation exchange chromatography (CEX) settings. These results support the integration of detergent treatment with a bind‐elute chromatography step, thereby simplifying the virus inactivation process and making it suitable for both batch and continuous manufacturing operations.

## 2 Materials and Methods

### 2.1 Detergent and Assay

Laureth 9 (L9), also known as Brij‐L9 or Virodex TXR‐1, was purchased from Croda International Plc (USA). The concentration of L9 was measured by high‐performance liquid chromatography coupled with evaporative light scattering detection (HPLC‐ELSD), based on previously described procedures (Gaber et al. 2014). The detection limit of this assay was 0.004% (w/v). L9 solutions at a concentration of 0.1% (w/v) in Tris‐based buffers or 10% (w/v) in water were stable for at least 6 months at room temperature, based on detergent content measurements.

### 2.2 Model Virus and Assay

XMuLV infectivity was measured using a plaque assay as described previously (Cai et al. 2019). Briefly, samples were serially diluted and incubated with indicator PG‐1 cells, obtained from American Type Culture Collection (Gaithersburg, MD, USA), to observe plaques. Virus titer (pfu/mL) was calculated based on the number of plaques and the original sample volume. XMuLV gc was measured using a TaqMan real‐time quantitative polymerase chain reaction (RT‐PCR) assay. The assay determines gc by comparing the PCR cycle threshold (Ct) values to a standard curve generated from known nucleic acid quantities. The correlation between virus RNA quantification and infectivity has been previously established using a serial dilution of XMuLV stock (Shi et al. 2004) or viral clearance test articles spiked with multiple viruses (Lute et al. 2009). The PCR has a limit of quantitation of 1 gc/µL (100% detection) with a 6‐log dynamic range and linearity of *r* ^2 ^> 0.99. All error bars in the figures represent 95% confidence intervals, including plaque assay and PCR, and assay variation is on the order of ± 0.5 log (ICH 2023).

### 2.3 Model Protein Molecules

Five IgG1 monoclonal antibodies (mAb1 to mAb5) were used as model proteins, produced from Chinese hamster ovary cells at AstraZeneca. The molecular weights ranged from 146 to 150 kDa, and the isoelectric points ranged from 7.3 to 8.8.

### 2.4 Impact of Matrix Composition on Detergent‐Based Virus Inactivation

To detect any impact from different product concentrations or impurities on detergent‐based virus inactivation, permeate materials from Day 1 and Day 14 of a perfusion bioreactor containing mAb1 were tested. The mAb1 concentration was 0.06 mg/mL for the Day 1 material and 2.76 mg/mL for the Day 14 material, a 46‐fold difference. After spiking each sample with approximately 8 logs of XMuLV in a beaker, the materials were treated separately with either 0.1% or 0.01% L9 detergent. Temporal samples were taken from 30 seconds to 10 minutes and quenched by a 1:50 dilution with neutral Tris buffer before measuring virus infectivity. Spiked hold control samples containing 0.002% or 0.0002% L9 were kept and tested at the end of the experiment to mimic residual virus inactivation after sample quenching. The reaction temperature was maintained at 15°C using a water bath.

### 2.5 Chromatography Setup for Virus Inactivation Using Detergent Wash

Prepacked HiScreen MabSelect Sure Protein A columns (Cytiva) were used for the initial experiments, and 1.0 × 10 cm columns of mixed resin containing 90% MabSelect Sure and 10% heparin agarose (Cytiva) were used to capture more virus in subsequent experiments. HiTrap CaptoS ImpAct (Cytiva) columns (1.6 × 5 cm in combined height) were used for the CEX experiments. The column runs were performed at ambient temperature. An AKTA Pure 25 system (Cytiva) was used to deliver the chromatography method with Unicorn software version 7.4.

The harvest cell culture fluid (HCCF) for loading the AC columns was spiked with XMuLV at a ratio of less than 1% (v/v) to achieve approximately 8 logs of infectivity. The AC columns were equilibrated with a neutral Tris‐buffer and loaded to column capacity. Next, 5 column volumes (CVs) of a wash buffer containing 0.1% detergent L9 were applied. The protein A column was not eluted to avoid any confounding virus inactivation effect from the low pH elution buffer. Instead, the resin beads and associated product were collected as one output sample and quenched before analysis. Control testing was performed to confirm no impact of resin beads on both PCR and plaque virus assays.

The CEX load material was spiked using the same method as described for the AC step. The CEX columns were equilibrated with an acetate buffer and loaded to column capacity. Next, 3 CVs of a wash buffer containing 0.1% L9 detergent were applied. The product was eluted using a buffer with slightly higher pH and increased salt, followed by a 1 M NaCl high salt strip. A combined product and strip sample was designated as the output sample in the CEX setting.

Control experiments were performed alongside using the same chromatography methods, but without detergent in the wash buffer, for the AC and CEX steps.

### 2.6 Distinguishing Virus Inactivation From Virus Removal

The overall net inactivation log reduction value (LRV) when using a post‐load wash with detergent was calculated using Equation 1:

(1) $$\mathrm{LRV}={\left[\log\frac{L}{\mathrm{gc}}-\log\frac{O}{\mathrm{gc}}\right]}_{\mathrm{with}\;\mathrm{detergent}}-{\left[\log\frac{L}{\mathrm{gc}}-\log\frac{O}{\mathrm{gc}}\right]}_{\mathrm{without}\;\mathrm{detergent}}$$

where “L” stands for the total virus infectivity in the spiked load material applied to the chromatography columns, and “O” stands for the total infectivity of the output sample, both values are expressed as plaque forming unit (pfu). For both AC and CEX, the output sample consists of resin‐bound virus fractions. The virus population is expressed as gc based on PCR results. The ratio of $\frac{L}{\mathrm{gc}}\mathrm{or}\frac{O}{\mathrm{gc}}\;$is the virus population normalized infectivity (VPNI), paralleling the concept used in epidemiology for the surveillance of virus prevalence between different communities of varying human populations (Amman et al. 2022; Yaniv et al. 2021). The second term in the Equation 1 corresponds to the baseline inactivation from the control experiment without detergent. The contribution from a baseline confounding mechanism was considered insignificant if the second term was less than one log, in which case the second term was omitted. In the current investigation, all infectivity test results were normalized to a common virus population of 10 billion gc, which correlates to about 8 logs of virus infectivity in a typical spiked load sample.

### 2.7 Process Performance and Product Quality Impact With Detergent Use

Four AC processes for mAb purification were evaluated with or without 0.3% L9 wash to detect the impact on process performance and product quality. In addition, CEX resins were washed with a buffer containing 1% L9 for 48 h or exposing 300 CVs of 0.1% L9 wash to simulate 100 cycles of column use. Process performance and product quality were evaluated by measuring process yield, protein concentration, pH, conductivity, and impurity profiles, including host cell protein (HCP), DNA, product monomer, and aggregates. HCP was measured using an ELISA‐based immunoassay, DNA was measured by PCR, and protein monomers and aggregates were analyzed by high performance size exclusion chromatography. Product profiles were compared to detect impact for these chromatography methods with and without detergent.

## 3 Results and Discussion

### 3.1 XMuLV Inactivation in Solution

#### 3.1.1 Rapid XMuLV Inactivation in Cell‐Free Permeate From a Perfusion Bioreactor

**Figure 2:**
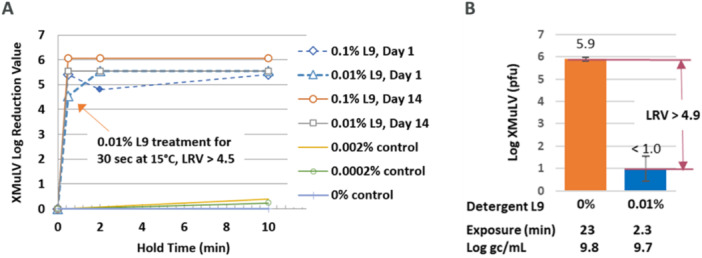
Rapid XMuLV Inactivation using L9 in solutions. (A) Inactivation time course of XMuLV spiked cell culture permeate containing mAb1, collected from either Day 1 or Day 14 of a perfusion bioreactor. Treatments with 0.1% and 0.01% L9 were evaluated alongside controls. Open symbols indicate virus inactivation below the limit of detection. (B) XMuLV inactivation in the wash buffer (mobile phase) of AC. The load material (mAb2) was not spiked. Instead, XMuLV was spiked into the AC wash buffer and delivered to the column while mixing in L9 for virus inactivation.

Treating HCCF with 0.1% L9 was previously shown to result in rapid inactivation of XMuLV to below the limit of detection within 1 min (Hunter et al. 2022). The current investigation reproduced similar results using a different mAb (mAb1) and at a lower L9 treatment concentration of 0.01%, as described in Section 2.4. Consistent levels of XMuLV inactivation, with a minimum LRV greater than 4.5 within 30 s were observed (Figure 2A).

The non‐ionic detergent L9 exists in equilibrium between monomers and micelles in solution, with a critical micelle concentration of 0.01% (Croda 2023). The rapid and robust XMuLV inactivation at 0.01% L9 concentration suggests that detergent monomers contribute to virus inactivation, as previously proposed (Negi et al. 2024; Farcet et al. 2023). The demonstration of robust XMuLV inactivation at a lower L9 concentration of 0.01% provides significant flexibility for process design and control.

#### 3.1.2 Inactivation of Virus During Passage Through an AC Column in the Mobile Phase

To study virus inactivation in the mobile phase of AC, a column was first loaded with mAb2 without a virus spike. Subsequently, XMuLV was spiked into a post‐load wash buffer containing 1 M NaCl. In the virus inactivation arm of the experiment, the spiked wash buffer was delivered to the column with L9 mixed in to achieve a final concentration of 0.01% L9, with a short exposure time at a fast flow rate. The L9 exposure in the mixer prior to the column was 9 s. A control experiment arm, conducted without detergent, used a slower flow rate to provide a longer exposure time (Figure 2B). The virus population in the samples collected from both arms was similar, as measured by PCR for gc number. XMuLV was inactivated to below the limit of detection (LRV > 4.9) in the presence of 0.01% L9, despite the shorter exposure time relative to the control arm. These results suggest that neither 1 M NaCl nor resin prevented virus inactivation, assuming any live virus flowed through the column in the mobile phase, effectively functioning as a tubular reactor. Inactivation can also occur in the pre‐column mixer, which is not differentiable with the current method.

### 3.2 XMuLV Inactivation Using AC Wash

#### 3.2.1 Inactivation of Resin Bound Virus

Results presented in Section 3.1 confirmed rapid virus inactivation using L9 in solution and in the AC mobile phase. Viruses can also associate with the stationary phase (resin) of a chromatography system, which needs further evaluation. Experiments to demonstrate on‐column, resin‐bound virus inactivation comprised three stages: (1) determination of the number of CVs required for equilibration with buffer containing 0.1% L9; (2) measurement of the capability of the detergent wash to inactivate bound virus; (3) control experiments conducted in the absence of detergent to demonstrate full recovery of infectivity.

#### 3.2.2 AC Operation and Detergent Treatment

**Figure 3:**
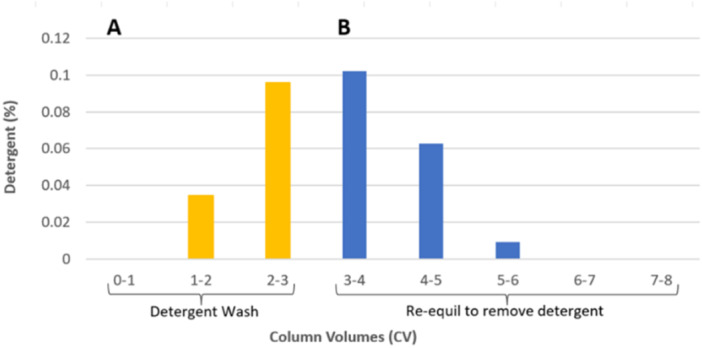
Detergent concentration changes during AC. (A) Detergent L9 concentration reached full resin equilibration after 3 CVs. (B) Detergent L9 was removed after 4 CVs of re‐equilibration buffer without detergent.

Prior to the virus spiking experiment, the volume of L9 wash buffer needed to equilibrate the AC column was determined by measuring the detergent content in the outflow. Columns were considered equilibrated with detergent when the concentration of L9 in the column outflow matched that of the wash buffer, alongside comparable pH and conductivity values. The system was challenged with 3 CVs of AC wash buffer containing 0.1% L9, which was adequate to equilibrate the column after 3 CVs (Figure 3A). Subsequently, L9 was removed by chasing with a non‐detergent re‐equilibration buffer, requiring 4 CVs to reduce L9 to below the limit of detection of the assay (Figure 3B).

**Figure 4:**
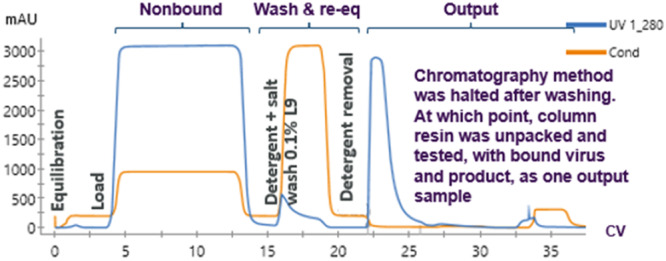
Conceptual illustration of virus inactivation using detergent L9 wash in AC. After loading XMuLV‐spiked HCCF, 0.1% L9 was applied for 5 CVs as a wash to inactivate the virus. The output sample comprised bound virus and product.

The following AC experiments with HCCF load included 5 CVs of 0.1% L9 wash, with a residence time of 2.4 min per CV. This translates to a 4.8 min of exposure time to 0.1% L9 from 2 CVs of wash, after 3 CVs required for equilibration. The column was then chased with equilibration buffer before collecting the output sample (Figure 4).

#### 3.2.3 XMuLV Removal During AC

**Figure 5:**
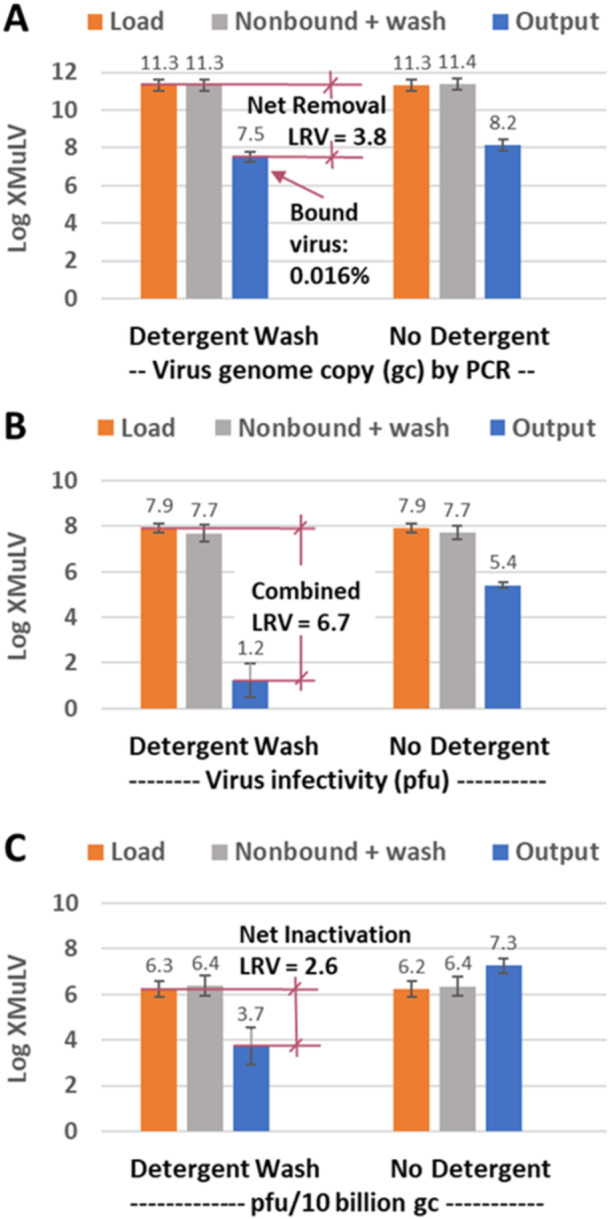
XMuLV Inactivation using 0.1% L9 detergent wash in AC. (A) Virus gc distribution measured using PCR. (B) XMuLV infectivity distribution measured using plaque assay. (C) Net virus inactivation LRV by L9 wash as measured using VPNI in pfu/10 billion gc.

Figure 5 summarizes the distribution of XMuLV gc (Figure 5A) and infectivity (Figure 5B) across the AC fractions, including load, non‐bound and wash, and output, with or without L9 wash. Figure 5A shows that virus gc was primarily recovered in the non‐bound and wash fraction. Only a small amount of the spiked XMuLV gc was detected in the output sample corresponding to the resin‐bound virus. This small amount represents just 0.016% of the spiked virus in the load material, thereby limiting the ability to measure the extent of virus inactivation. The net virus removal LRV with the L9 wash for the AC step was 3.8, while slightly lower removal was observed in the absence of detergent wash (LRV = 3.1).

#### 3.2.4 XMuLV Inactivation During AC With Detergent Wash

Figure 5B shows the distribution of infectivity, including both inactivation and removal mechanisms, across the same chromatography fractions. When the wash buffer containing 0.1% L9 was applied to the affinity column with bound product and virus, XMuLV was inactivated to a very low level of 1.2 log pfu. An LRV of 6.7 was calculated by comparing it with 7.9 log pfu of virus infectivity in the load (Figure 5B, “Detergent Wash”). Furthermore, the 1.2 log pfu is dramatically lower than the output sample of 5.4 log pfu from the control experiment without L9 wash (Figure 5B, “No Detergent”), confirming the role of L9 in the post‐load wash inactivation over the bound virus.

#### 3.2.5 Applying VPNI to Differentiate Net Virus Inactivation Versus Net Virus Removal

**Figure 6:**
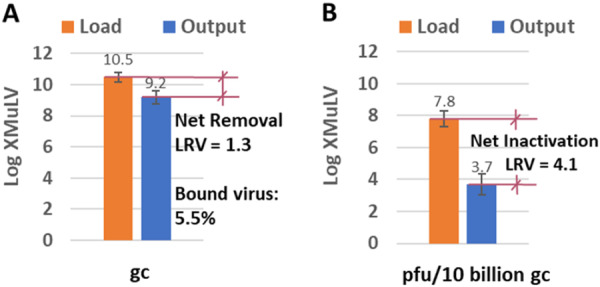
Detergent wash virus inactivation for AC with enhanced virus immobilization using a mixed resin of 90% protein A and 10% heparin. (A) More XMuLV was immobilized to the resin (5.5%). (B) Net virus inactivation by 0.1% L9 wash determined using VPNI.

When applying VPNI to determine net inactivation, an LRV of 2.6 was calculated (Figure 5C). This low LRV is likely influenced by the very small percentage of bound virus in the system (0.016%, Figure 5A). To circumvent this problem, 10% heparin agarose resin was included with the Protein A resin to increase virus capture within the chromatography packed bed environment (Segura et al. 2008). This led to an increase in bound virus by more than 300‐fold (about 5.5% of the spiked virus in the load), and a measured net inactivation LRV of 4.1 (Figure 6).

In Figure 5C, the no‐detergent output shows high VPNI as expected, but it also exceeded the load. Given the use of two complex assays, a variability of ± 0.5 log per assay (ICH 2023) may account for this. Importantly, persistence of infectivity is demonstrated relative to the reduced infectivity observed in the detergent wash arm, and this key conclusion remains unchanged.

### 3.3 XMuLV Inactivation Using CEX Wash

CEX is commonly used in bind‐elute mode to remove impurities and putative viruses. Several features of CEX make it a useful tool for investigating virus inactivation on the column. First, the equilibration pH is typically acidic (4–6), which is lower than the isoelectric points of most mAbs and retroviruses (Strauss et al. 2009). As a result, both mAbs and retroviruses are positively charged and captured by the negatively charged CEX resin. Second, viruses bind to the resin more strongly than proteins because of their larger size and multiple binding sites. This allows the protein product to be eluted first using a moderate salt concentration, while the viruses remain bound to the resin. Third, the virus can then be recovered with a high‐salt strip, such as 1 M NaCl, without loss of infectivity (Cai et al. 2019). Finally, detergent L9 does not bind to agarose‐based CEX resin, enabling easy removal after viral inactivation and prior to product elution.

#### 3.3.1 CEX Operation and Detergent Treatment

**Figure 7:**
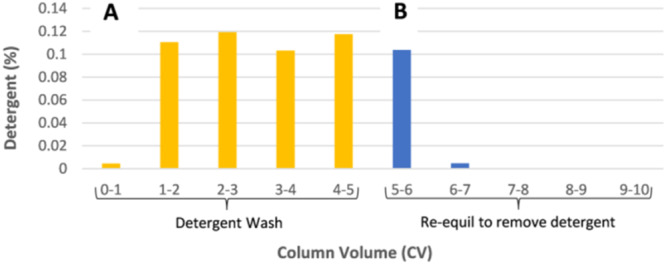
Detergent concentration from the outlet of a CEX column. (A) During a post‐load wash containing detergent L9, the concentration reached 0.1% with complete resin equilibration after 2 CVs. (B) Re‐equilibration buffer without detergent reduced L9 to below the limit of detection after 3 CVs.

**Figure 8:**
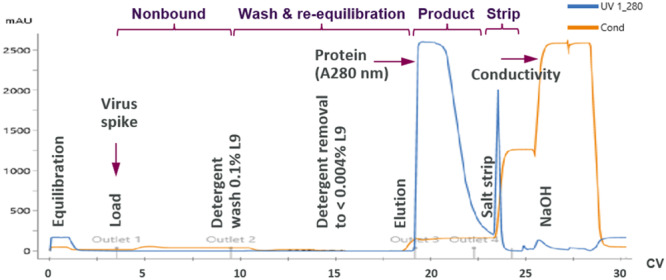
CEX chromatogram with detergent wash for virus inactivation following product load. A detergent wash of 3 CVs was applied to inactivate the virus. The detergent was subsequently removed with 5 CVs of re‐equilibration buffer.

The agarose‐based resin Capto S ImpAct reached the target L9 concentration of 0.1% after applying only 2 CVs of the L9 wash buffer (Figure 7A). Subsequently, L9 was removed by re‐equilibrating the system with a buffer lacking detergent; after just 3 CVs, the L9 concentration in the elution was reduced to below 0.004% (Figure 7B). Based on these results, a protocol was implemented consisting of 3 CVs of L9 wash, followed by 5 CVs of re‐equilibration, with a residence/exposure time of 3.4 min per CV. This exposure time is significantly longer than the 30 s required to inactivate more than 4.5 logs of XMuLV in solution (Figure 2A). A representative CEX chromatogram using Capto S ImpAct is illustrated in Figure 8.

#### 3.3.2 XMuLV Removal During CEX

**Figure 9:**
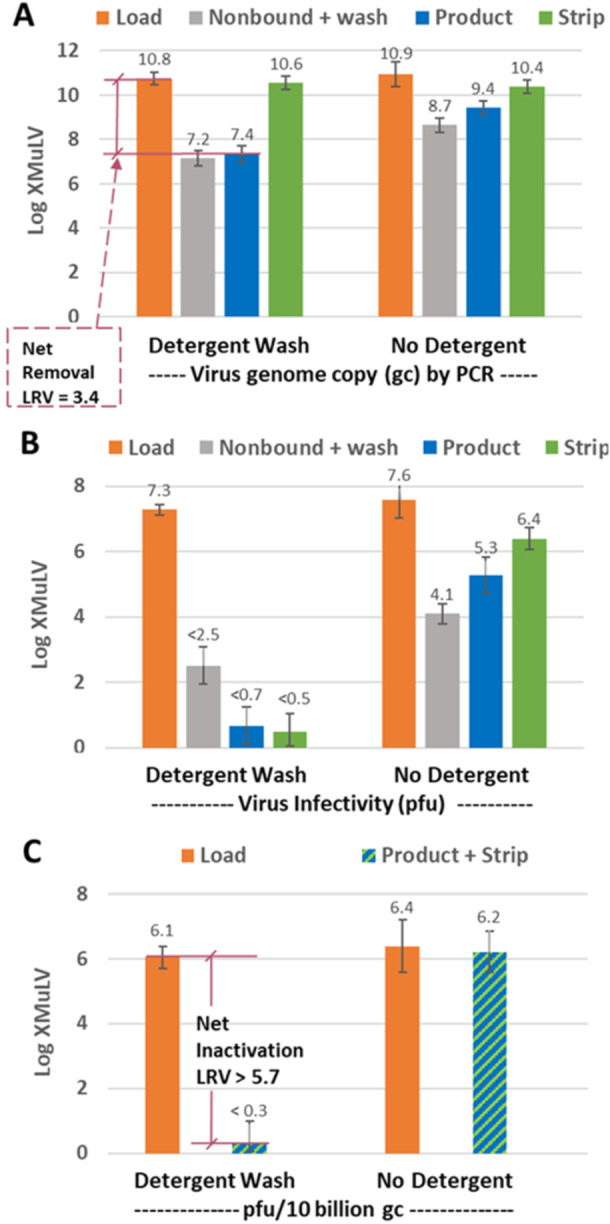
XMuLV Inactivation using 3 CVs of 0.1% L9 detergent wash in CEX. (A) Virus gc measured using PCR. (B) XMuLV infectivity measured using plaque assay. (C) VPNI in pfu/10 billion gc, comparison of load versus a combined output sample consisting of product and strip.

Figure 9 summarizes the distribution of XMuLV gc (Figure 9A) and infectivity (Figure 9B) across the Capto S ImpAct CEX process, with or without the L9 wash, determined from the same sample. Little of the spiked XMuLV gc was detected in the non‐bound, wash, and product fractions (Figure 9A). Instead, the virus gc was primarily recovered in the salt strip. This indicates near complete capture and strong association of XMuLV with the CEX resin, regardless of detergent wash (Figure 9A). Accordingly, the mass balance was established mostly between the load and strip. The net virus removal LRV with the L9 wash was 3.4 as measured by the gc differential between the load and product. Lower XMuLV removal from the product was observed for CEX in the absence of detergent wash (LRV = 1.5).

#### 3.3.3 XMuLV Net Inactivation Using L9 Wash for CEX

When the column was washed with L9 after loading, XMuLV was inactivated to below the limit of detection for both the product and salt strip fractions (Figure 9B, “Detergent Wash”). This is in remarkable contrast to the infectious virus recovered in the absence of L9 wash in the otherwise identical CEX setting (Figure 9B, “No Detergent”).

Using VPNI, a net XMuLV inactivation LRV of > 5.7 was observed with the detergent wash arm (Figure 9C). In contrast, XMuLV inactivation was minimal in the absence of detergent wash, where the spiked virus infectivity was quantitatively recovered from the combined product + strip sample. Robust virus inactivation with L9 wash was reproduced using a polystyrene‐based resin, Poros 50HS (data not shown). These results specifically demonstrate the presence of virus genome copy in the samples while losing infectivity only after detergent treatment.

The PCR measurement of gc captures both encapsulated and free viral RNA, which could potentially affect VPNI calculation if abundant free RNA binds the resin differently than virus particles. However, both RNA (encapsulated or free) and infectivity recover in the CEX strip (Figure 9A, green bars). Minimal gc changes after detergent wash suggest that free RNA is either scarce or not selectively binding, and the VPNI calculation remains objective.

### 3.4 Impact of Detergent Use on Process Performance and Product Quality

Product quality impact was evaluated using the AC method with and without detergent, for four mAbs. To challenge the process, the detergent content in the post‐load wash was increased to 0.3% in the detergent treatment arm. No negative impact trend was observed when comparing eluted product protein concentration, pH, conductivity, yield, HCP, DNA, product monomer, and aggregates between the products with and without detergent exposure (Table 1). With detergent wash, lower HCP levels were noted for mAb 4 and mAb 5, and lower multimer and fragment percentages were observed for mAb 3 and mAb 5, suggesting a potential project‐specific advantage of using detergent in post‐load wash for impurity removal.

**Table 1:** Comparison of AC products with or without 0.3% L9 wash.

Entity	mAb 2		mAb 3		mAb 4		mAb 5	
0.3% L9 wash	No	Yes	No	Yes	No	Yes	No	Yes
Product (mg/mL)	24.8	24.1	24.9	24.9	17.2	17.4	21.5	22.9
pH	4.6	4.6	4.6	4.6	4.7	4.4	4.7	4.7
Conductivity (mS/cm)	0.69	0.72	0.67	0.71	0.89	0.73	0.89	0.94
Yield (%)	98	101	88	87	98	94	95	100
HCP (ng/mg)	146	155	206	223	2563	1335	117	84
DNA (ng/mg)	0.4	0.3	1.4	1.9	2.2	2.3	0.2	1
Monomer (%)	99.4	99.4	81.5	91.2	90.6	90.6	87.3	92.1
Multimer and fragment (%)	0.6	0.6	18.5	8.8	9.4	9.4	12.7	7.9

Two experiments were performed to assess the impact of exposing 1% L9 for 48 h or simulating 100 cycles of 0.1% L9 wash on the integrity and performance of CEX. Under these conditions, a negligible impact on process performance or product quality was observed for the attributes mentioned above (data not shown). Long‐term product stability will be assessed for each specific project following the implementation of detergent wash in the future.

## 4 Conclusion

This investigation demonstrates that virus inactivation can be achieved using an L9 detergent‐containing post‐load wash in both AC and CEX settings. XMuLV was inactivated both in its free form (suspended in the mobile phase) and when the virus is bound to a chromatography resin. The findings provide evidence that XMuLV is not protected by the resin or column configuration, and that detergent L9 can readily access and inactivate the virus. This conclusion aligns with the mechanism of inactivation via virus envelope deformation and viral protein solubilization, facilitated by the detergent's small size (~0.6 kDa) compared with a mAb (~140 kDa) and the much larger size of a virus. While the results align with previous reports of virus inactivation in chromatography systems (Bolton et al. 2015; Polasek et al. 2023), this study contributes further mechanistic insight through the inclusion of additional controls. In particular, the introduction of VPNI calculation enables quantification of net virus inactivation LRV and distinguishes it from virus removal. The CEX wash step inactivated XMuLV to below the limit of detection and is therefore appropriate for inclusion in a regulatory submission, whereas the AC step requires further development. In summary, these findings provide a simplified approach to robust virus inactivation capacity, as required by ICH Q5A R2 (ICH 2023), thereby supporting more efficient manufacturing processes.

## Author Contributions

Study concept and design: Kang Cai, Gisela Ferreira, Chris Afdahl, Etienne Utiger, and Jennifer Anderson. Study execution: Etienne Utiger, Rayan Zamat, and Yeldys De Armas. Manuscript preparation: Kang Cai, Gisela Ferreira, Etienne Utiger, Chris Afdahl, Jennifer Anderson, and Rayan Zamat.

## Conflicts of Interest

The authors declare no conflicts of interest.

## Data Availability

The data that support the findings of this study are available from the corresponding authors upon reasonable request.
